# Manual action verbs modulate the grip force of each hand in unimanual or symmetrical bimanual tasks

**DOI:** 10.1371/journal.pone.0192320

**Published:** 2018-02-05

**Authors:** Ronaldo Luis da Silva, David Labrecque, Fátima Aparecida Caromano, Johanne Higgins, Victor Frak

**Affiliations:** 1 Faculté des Sciences, Université du Québec à Montréal, Quebec, Canada; 2 Centre de recherche interdisciplinaire en réadaptation (CRIR), site de l’Institut de Réadaptation Gingras-Lindsay-de-Montréal (IRGLM) du CIUSSS Centre-Sud-de-l'Île-de-Montréal, Quebec, Canada; 3 Laboratory of Physical Therapy and Behaviour, Department of Physical Therapy, Speech and Occupational Therapy, University of São Paulo Medical School, São Paulo, Brazil; 4 École de Réadaptation, Faculté de Médecine, Université de Montréal, Québec, Canada; University of Exeter, UNITED KINGDOM

## Abstract

Manual action verbs modulate the right-hand grip force in right-handed subjects. However, to our knowledge, no studies demonstrate the ability to accomplish this modulation during bimanual tasks nor describe their effect on left-hand behavior in unimanual and bimanual tasks. Using load cells and word playlists, we evaluated the occurrence of grip force modulation by manual action verbs in unimanual and symmetrical bimanual tasks across the three auditory processing phases. We found a significant grip force increase for all conditions compared to baseline, indicating the occurrence of modulation. When compared to each other, the grip force variation from baseline for the three phases of both hands in the symmetrical bimanual task was not different from the right-hand in the unimanual task. The left-hand grip force showed a lower amplitude for auditory phases 1 and 2 when compared to the other conditions. The right-hand grip force modulation became significant from baseline at 220 ms after the word onset in the unimanual task. This moment occurred earlier for both hands in bimanual task (160 ms for the right-hand and 180 for the left-hand). It occurred later for the left-hand in unimanual task (320 ms). We discuss the hypothesis that Broca’s area and Broca’s homologue area likely control the left-hand modulation in a unilateral or a bilateral fashion. These results provide new evidence for understanding the linguistic function processing in both hemispheres.

## Introduction

In their 2010 article, Frak and colleagues [1] found that linguistic stimulation modulates the right-hand grip force. By means of small load-cells, they evaluated the grip force behavior exerted by subjects while they listened to a list of words containing non-action nouns and a manual action-verb repeated a controlled number of times or a list of action verbs with a non-action noun as the keyword. They compared the mean grip force variation exerted along 800 ms following the start of the word. They found significantly different grip force variations between action verbs and non-action nouns within the 260 and 430 ms time interval. Since this modulation was word class-specific, the authors could affirm that the modulation followed the linguistic stimuli.

The left hemisphere dominance for word generation is extensively recognized in the scientific literature [2]. Some authors consider Broca’s area to be involved with syllabic representations [3], while others consider that it is implicated in representation of the full articulatory processing [4], sharing different levels of coordination for the word production with the Brodmann area 6 [5, 6]. Stout and Chaminade [7], illustrating the link between speech and tool use, have shown a massive network connecting the Wernicke area, parietal inferior lobe, premotor cortex, and Broca’s area. Recent studies have demonstrated that Broca's area communicates with the left supplementary motor area by means of the frontal aslant tract (FAT); and with the left dorsal premotor cortex by means of U-fibers that run over the FAT surface [8]. These findings denote the close relationship between Broca's area and the left primary motor cortex (M1) control center and support its influence on motor activity due to linguistic stimulus.

The left hemisphere dominance for right-handed subjects is well established for word production as well as for word comprehension [9]. Broca’s area is considered as a key center for language processing and for activation of the cortical network that accounts for speech production [10]. Also, from a purely motor standpoint, there is a left hemispheric dominance for motor control of the hand. While the left M1 shows the same level of activation during right unimanual and symmetrical bimanual activities, the activation of the right M1 exhibited during left unimanual activity is significantly reduced during symmetrical bimanual activity [11]. The symmetrical bimanual movements seem to be controlled by the left M1, which defines the parameters for the left muscles through the ipsilateral corticospinal tract and generates a transcallosal inhibition of its contralateral counterpart without completely excluding its control over the left-hand [11, 12]. Even left unimanual activity shows some degree of left asymmetry, since control of the right M1 is exerted simultaneously by the right supplementary motor area and the right dorsal premotor cortex as well as by the left supplementary motor area [13].

Therefore, linguistic processing and motor control demonstrate left hemisphere dominance. The results found by Frak et al. [1] corroborate with previous studies demonstrating the relationship between linguistic and motor functions. However, no study has investigated the influence of language on the left-hand motor function during unimanual or bimanual tasks. The purposes of this study are to investigate if linguistic stimulation, by means of manual action verbs, modulates right-hand grip force during symmetrical bimanual task and the left-hand grip force in both unimanual and symmetrical bimanual tasks, and if so, to evaluate the differences among these modulations.

## Materials and methods

### Ethics statement

Ethics approval was obtained from the Research Ethics Board–REB–of the *Centre de recherche interdisciplinaire en réadaptation du Montréal métropolitain* (Centre for Interdisciplinary Research in Rehabilitation of Greater Montreal–CRIR).

### Participants

Participants were recruited through public advertisements in centers related to the Brazilian community in Montreal or were directly invited by other volunteers. Every participant was invited to answer the Edinburgh Handedness Inventory. An 80% right-handedness cut-off was defined as eligibility criteria for assuring right-handedness consistency [14–15]. Participants self-reported that they did not have neurological or musculoskeletal disorders nor deficits in cognitive or motor skills. Ten men ranging in age from 23 to 47 years (32.3 ± 6.6) and 20 women from 22 to 51 years (35.1 ± 8.1) with 12 or more years of Portuguese-language schooling signed the consent form and participated in this study.

### Stimuli

A list of verbs and nouns selected from a previous study [1] were translated from French into Portuguese and recorded in a studio in MP3 format at 44.1 kHz and 32 bits with a male voice. Selected action verbs described manual actions such as “write” or” scratch.” Selected non-action nouns described concrete objects or beings but never actions. Words that could be used as both nouns and verbs were excluded from the selection. Since there is not a reference work for word frequencies in Brazilian Portuguese, action verbs selection was made using the French parameters as a general guide. Mean word duration was 684 ms. These words were isolated and converted to WAV format at 16 kHz and 8 bits and then transferred to the management system. The system allowed for the selection of a keyword, and automatically identified its word class and generated a playlist containing 18 repetitions of this keyword distributed among 70 words from the other word class. In our study, keywords were always an action verb. Since we had 35 recorded non-action nouns, they were repeated twice in the playlist. The system divided the final playlist of a single session into two auditory blocks of similar length, thus the number of repetitions of the keyword in each block was variable. The interval between two consecutive words was 1000 ms, and the word sequence was randomized for each participant.

### Procedure

**Fig 1:**
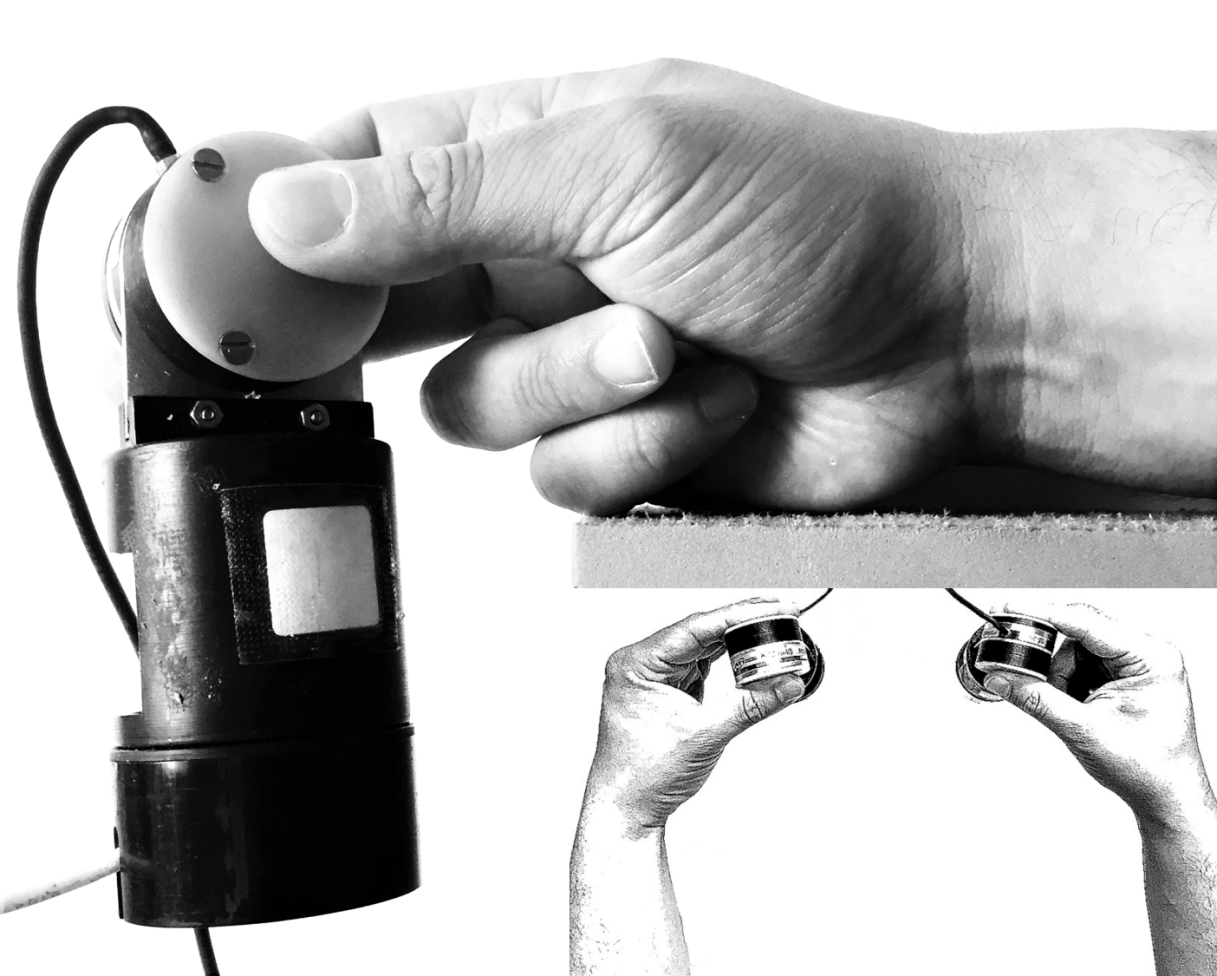
Grip force sensor held by means of a tri-digital pinch. Arm support extends to the fifth metacarpal head while the sensor remains hanging in the gap. The sketch in detail shows the symmetrical bimanual positioning and displays the neutral position of flexion/extension of the wrists.

Participants wore headphones and were seated comfortably at a table with a gap along its width, allowing for the positioning of the grip force sensors. Participants kept both forearms in a neutral position and supported from distal elbow to the proximal metacarpal region, with their fingers holding the sensors over the gap. Participants were told to grip the sensor using a tri-digital pinch, keeping the wrist in a neutral position of pronation/supination and flexion/extension and exerting a minimum of force, just enough to prevent dropping, as shown in Fig 1. Only the compression force Fz was evaluated, since a previous study found no differences between the obtained curves for Fz alone and the sum of the three-axis forces [1]. Participants were introduced to the system and the test procedure and trained in positioning of the upper limb and tri-digital pinch gripping. They could see the compression force intensity on a secondary screen. They were asked to increase and reduce their force intensity to find a comfortable grip force level, generally between 1.5 and 2.5 N. Since our device containing the sensor weighed 155 g and the slip-ratio limit was estimated to be less than 75 mN, this grip force level was high enough to prevent slippage [16]. They didn’t have access to the primary screen, and the secondary screen remained turned off during the entire test. All tests were applied by the same researcher.

The test was performed in a single day and was comprised of six sessions. Each session consisted of ten sequential steps: 1) hearing a keyword by the headphones, 2) grasping the grip force sensor, 3) holding it over the table gap, 4) closing the eyes, 5) listening to a playlist lasting approximately 80 seconds, 6) counting how many times the keyword was on the playlist (a strategy adopted to ensure the participant's attention to the task), 7) opening the eyes, 8) setting the grip force sensor down on the table, 9) reporting the number of repetitions of the keyword, and 10) waiting approximately one minute before repeating all the steps for the second block of the same session. The keyword was always an action verb and was different for each session. In summary, each participant heard 12 auditory blocks, each of 41–47 words, in which each sequence consisted of a variable number of presentations of the action-verb keyword and of the 35 non-action nouns. These words were randomly mixed with the constraint that a keyword could not be immediately repeated. The 12 auditory blocks performed six playlists, and the two auditory blocks of the same playlist were presented sequentially. Participants performed two sessions with the right hand, two sessions with the left hand and two sessions with both hands (symmetrical bimanual). When performing the symmetrical bimanual task, subjects held two grip force sensors, one for each hand. The six-session sequence was randomly determined for each participant before the test. Each session had its own distinctive keyword.

### Data acquisition

The output of the compression force was generated by a standalone F/T sensor system controller (ATI Industrial Automation, NC, USA), which can evaluate the force in the three Cartesian axes simultaneously. For the left-hand, the sensor was a 4 N/V (or 9.77 mN/bit) Mini40 SI-20-1, and for the right-hand was an 8 N/V (or 19.53 mN/bit) Mini40 SI-40-2. In this study, the compression force Fz was recorded at 1 kHz. The signals were recorded with a PCI-6024E A/D card (National Instruments, TX, USA) and the playlists were delivered through a D/A channel of a PCI-MIO-16E-4 card connected to the headphones. Although the grip sensor was different for each hand, the analogic output from the acquisition card has ensured that there was no difference between the grip sensor resolution in this study. Card synchronisation assured that the output of the digitised playlists automatically triggered the acquisition of grip data.

### Data analysis

Prior to data analysis, each signal component was filtered at 15 Hz with a fourth-order, zero-phase, low-pass Butterworth filter, allowing almost 95% of signal energy conservation. Data were then segmented from 300 ms before to 1000 ms after the onset of the word. The mean of the signal 200 ms before the word started was used to normalize the data for that word. If the signal variation during 200 ms before and 800 ms after word started was greater than or equal to 200 mN, or developed at a rate greater than 100 mN in a 100 ms interval, data relative to this word were rejected [16]. When 30% of the words for a single condition were rejected, all the participant data for the four conditions were rejected. According to this method, three men and two women were excluded from the analysis by the end of the normalization data. For each keyword repetition, data from 200 ms prior to the word onset were averaged, and this value was used to normalize values for the following 1000 ms. We adopted this procedure because our objective was to evaluate the force variation along the time frame following the word onset. A further foundation for these settings is provided in Nazir et al. [16].

Right-hand grip force modulation as a language-induced motor activation was investigated in several studies [1, 16, 17]. Over time, the neurophysiological model of spoken sentence comprehension described by Friederici [18] has become the basis for analyzing grip force modulation arising from language stimulation. This model describes three phases. Phase 1 takes place approximately 100–300 ms after word onset and generates information about syntactic structure. Phase 2 hosts lexical-semantic and morphosyntactic processes in a broader analysis along the next 200 ms (300–500 ms), and phase 3, starting near 500 ms after word onset, integrates and reanalyzes all the information from the previous phases. Previous studies have found a significant increase in grip force during each phase when compared to the baseline, defined as the mean grip force variation for the 200 ms prior to word onset [1, 16]. Data were organized in four conditions according to the hand used and the manual task (unimanual or bimanual): symmetrical bimanual right-hand (SBR), symmetrical bimanual left-hand (SBL), unimanual right-hand (UR) and unimanual left-hand (UL). Since the comparison between non-action nouns and action verbs is currently well documented [1, 17], only the keywords were analysed in this study.

For each participant, a mean value was obtained from the keyword repetitions of both sessions for each condition for the baseline and the three auditory processing phases of Friederici. This dataset was used to characterize the occurrence of grip force modulation for the right-hand and the left-hand in a unimanual and a symmetrical bimanual task. Although the literature already provides information about language-induced right-hand grip force modulation in a unimanual task, our right-hand data were used as for comparison.

The occurrence of grip force modulation was defined as a significant increase in grip force between the baseline and one or more auditory processing phases. One-way repeated measures ANOVAs were conducted for each condition comparing the baseline to the three phases following the stimulus, as well the phases with each other. These were followed by a post hoc LSD.

The characterization of the four conditions was made in two steps. First, a three-way repeated measures ANOVA was conducted using the within-subject factors TASK (unimanual vs. symmetrical bimanual), HAND (right vs. left) and PHASE (phase 1 vs. phase 2 vs. phase 3) to provide a full analysis of all possible interactions. Paired t-tests were made between conditions for which there was a significant main effect, two-by-two, for each phase comparing tasks (UR vs. UL and SBR vs. SBL), and hands (UR vs. UL and SBR vs. SBL).

The second step aimed to identify the moment at which the grip force became significantly different from the baseline. For this purpose, the first phase that was significantly different from baseline was divided into 50 ms time intervals for each participant. This analysis consisted of a one-way repeated measures ANOVA comparing the mean value for each time interval to baseline and has been followed by a post hoc LSD. The period between the word onset and this specific moment was defined as reaction time (RT), and it was identified for each of the four conditions. After defining the time interval containing the RT for each condition, we proceeded with an analysis with this time interval along with the preceding and following ones by dividing them into 10 ms micro-windows to better locate the RT using the same method.

## Results

### Occurrence of modulation

There was no difference between the baselines when compared by task or by hand (F _(1,24)_ = 0.092, *p* = 0.764 and F _(1,24)_ = 0.049, *p* = 0.826, respectively). The mean grip force for the three phases was significantly different from its respective baseline for UR, SBR, and SBL. For UL, only phases 2 and 3 were significantly different from baseline. For all conditions, phase 1 was significantly different from phases 2 and 3, but phase 2 was significantly different from phase 3 only for UL (Table 1). These findings support that there was a language-induced grip force modulation for the four studied conditions.

**Table 1:** Comparison between baseline and auditory processing phases by the condition.

		**Baseline / Phase 1**		**Baseline / Phase 2**		**Baseline / Phase 3**	
		*p*	*sig*	*p*	*sig*	*p*	*sig*
UR	F _(1.361,32.668)_ = 14.306	*0*.*030*	yes	*0*.*000*	yes	*0*.*001*	yes
UL	F _(1.653,39.680)_ = 15.168	*0*.*740*	no	*0*.*001*	yes	*0*.*000*	yes
SBR	F _(1.478,35.467)_ = 20.125	*0*.*007*	yes	*0*.*000*	yes	*0*.*000*	yes
SBL	F _(1.590,38.156)_ = 26.842	*0*.*027*	yes	*0*.*000*	yes	*0*.*000*	yes
		**Phase 1 / Phase 2**		**Phase 1 / Phase 3**		**Phase 2 / Phase 3**	
		*p*	*sig*	*p*	*sig*	*p*	*sig*
UR	F _(1.361,32.668)_ = 14.306	*0*.*000*	yes	*0*.*001*	yes	*0*.*074*	no
UL	F _(1.653,39.680)_ = 15.168	*0*.*000*	yes	*0*.*000*	yes	*0*.*033*	yes
SBR	F _(1.478,35.467)_ = 20.125	*0*.*000*	yes	*0*.*000*	yes	*0*.*122*	no
SBL	F _(1.590,38.156)_ = 26.842	*0*.*000*	yes	*0*.*000*	yes	*0*.*332*	no

SBR: symmetrical bimanual task, right-hand; SBL: symmetrical bimanual task, left-hand; UR: unimanual task, right-hand; UL: unimanual task, left-hand. Phase 1: 100–300 ms. Phase 2: 300–500 ms. Phase 3: 500–800 ms. Degrees of freedom following a Greenhouse-Geisser correction. One-way repeated measure ANOVAs significant at *p* < 0.001.

### Characterization by condition

The three-way repeated measures ANOVA found no main effect for the TASK factor (F _(1,24)_ = 2.713, *p* = 0.1126) and found a significant main effect for the HAND (F _(1,24)_ = 6.375, *p* = 0.0186) and PHASE factors (F _(1.298,31.142)_ = 83.902, *p* < 0.001). However, no significance was found for TASK*HAND (F _(1,24)_ = 0.028, *p* = 0.8674), TASK*PHASE (F _(1.192,28.604)_ = 0.531, *p* = 0.5028), and HAND*PHASE (F _(1.482,35.564)_ = 1.844, *p* = 0.1802) interactions between subjects, nor for the TASK*HAND*PHASE interaction (F _(1.189,28.529)_ = 0.089, *p* = 0.8107). Paired t-tests showed that for the two first phases, there was a significant difference between unimanual and symmetrical bimanual tasks for the left-hand (phase 1: t _(24)_ = 2.133, p < 0.05; phase 2: t _(24)_ = 2.864, *p* < 0.01), and between the left and right hand for the unimanual task (phase 1: t _(24)_ = 2.550, p < 0.05; phase 2: t _(24)_ = 2.228, *p* < 0.05).

**Fig 2:**
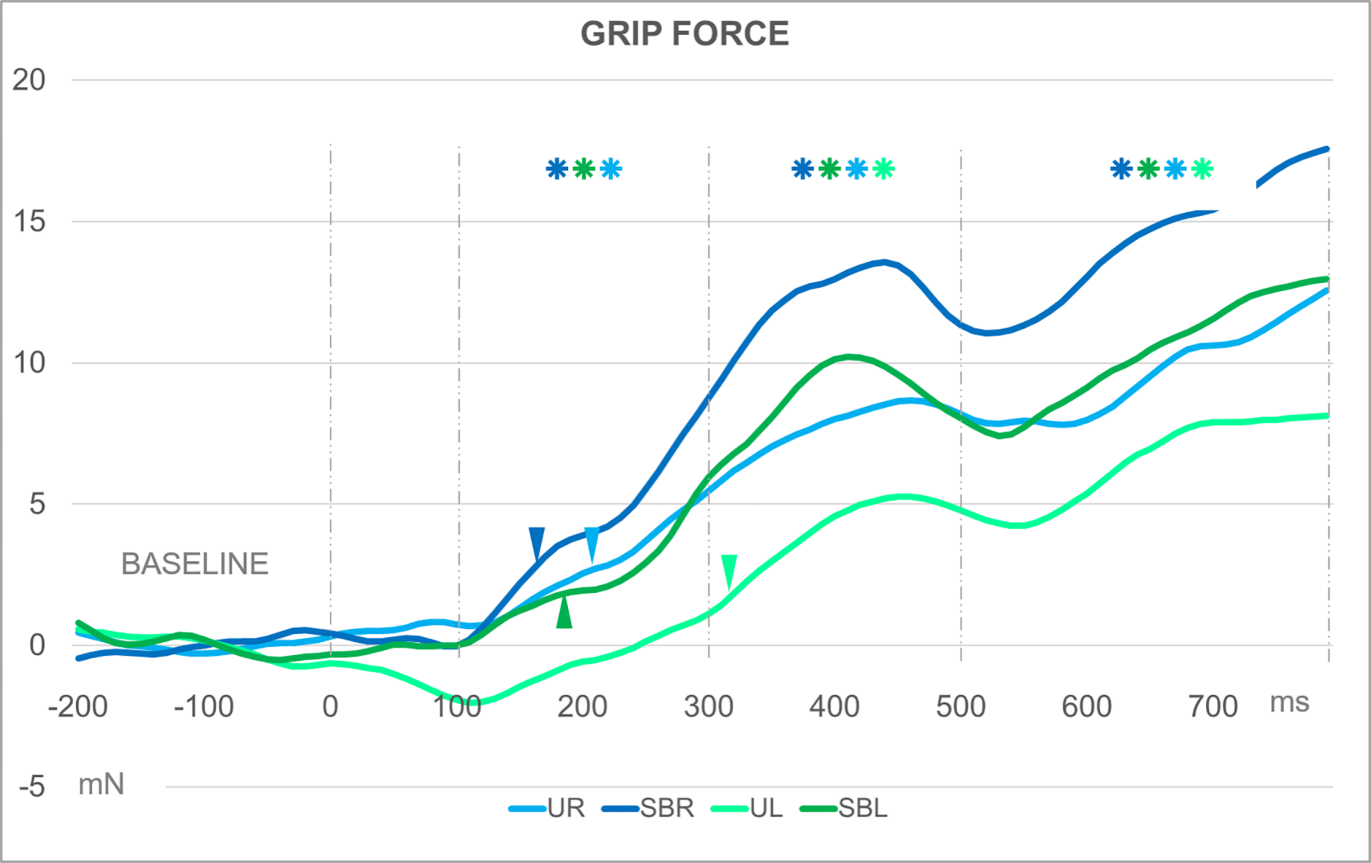
Grip force modulation and RT according to the tasks. Curves illustrating grip force modulation from -200 ms until the end of the third phase of the neurophysiological model of spoken sentence comprehension described by Friederici [18] compared to baseline and the moment in which the reaction time (RT) occurs (triangles). UR: unimanual task, right-hand; SBR: symmetrical bimanual task, right-hand; SBL: symmetrical bimanual task, left-hand; UR: unimanual task, right-hand. Blue curves used for the right hand and green curves for the left hand. Asterisks indicate a significant difference between baseline and the modulation. Asterisk color indicates the significant condition. Time in milliseconds after the word onset. Y-axis: grip force variation in millinewtons.

The one-way repeated measures ANOVAs conducted to identify the RT time interval by condition showed that the first significant increase in grip force for UR occurred at the 200–250 ms time interval [F (_1.359,32.616_) = 7.653, *p* = 0.027]. For UL, the RT was found at 300–350 ms [F (_1.867,44.812_) = 12.961, *p* = 0.015]. For both SBR and SBL, the RT was found at 150–200 ms [F (_1.705,44.678_) = 11.073, *p* = 0.030 and F (_1.842,44.203_) = 8.756, *p* = 0.006, respectively]. The micro-windows analysis found the RT for UR at 220–230 ms [F (_1.150,55.324_) = 4.682, *p* = 0.048] and for SBR at 160–170 ms [F (_1.252,46.361_) = 6.341, *p* = 0.030]. For the left hand, the RT for the symmetrical bimanual condition occurred within the 180–190 ms micro-window [F (_1.518,36.427_) = 3.157, *p* = 0.039], while the RT for the unimanual condition was found within the 320–330 ms micro-window [F (_1.152,21.964_) = 6.557, *p* = 0.030]. Modulation across time for the four conditions and the RT are graphically represented in Fig 2.

## Discussion

Applying Friederici’s model, the analysis of the four conditions showed that there was grip force modulation for all conditions, that is, the involuntary grip force modulation following auditory stimulation using a manual-action verb occurred for both hands in both studied tasks.

The right-hand showed a significant grip force increasing from baseline for the three auditory processing phases in the unimanual task. Comparing the phases to each other, phase 1 was different from phases 2 and 3, and phase 2 was not different from phase 3. The right hand showed the same results in the symmetrical bimanual task. No statistical difference was found when comparing the same phase of both tasks. Therefore, there is no doubt that the manual action verb modulated the grip force of the right hand during the symmetrical bimanual task, that is, the right-hand grip force modulation occurs in both tasks. Interestingly, the left hand presented the same results as the right hand, that is, the three phases were significantly different from baseline, phase 1 was different from phases 2 and 3, and phase 2 did not differ from phase 3. All the employed tests found no difference when comparing the same phase of both hands in the symmetrical bimanual task. It allows us to affirm that there is grip force modulation for the left-hand in a symmetrical bimanual task. Furthermore, these results show that the grip force modulation across the auditory processing phases for these three conditions–the right hand in both tasks and the left hand in the symmetrical bimanual task–is very similar.

The left hand in the unimanual task showed several differences compared to the other three conditions. Phase 2 was the first to show a significant difference from baseline, even though phase 1 was different from phases 2 and 3 as the other conditions. However, phase 2 was different from phase 3. According to these findings, the grip force of the left hand was also modulated by the linguistic stimulus, although this modulation became significant later relative to the other conditions. This is reinforced by the fact that UL phases 1 and 2 were significantly different from the same phases of the other conditions, showing a smaller amplitude of modulation.

The RT for the right hand in the unimanual task showed similar values to those found previously [1, 17] when comparing action verbs to non-action nouns. However, the RT analysis showed that modulation became significantly different from baseline earlier for both hands in the symmetrical bimanual task, that is, the symmetrical condition made the grip force modulation more quickly detectable. In the symmetrical bimanual task, the RTs for the left and right hand were very close, with the left-hand RT being only 20 ms longer. In the unimanual task, the left-hand RT took place at the 320–330 ms micro-window, 100 ms after the UR RT.

Thus, we can affirm that there is language-induced grip force modulation for the left-hand in a unimanual task, though it takes longer to become consistent enough to be statistically significant. When acting together, both hands show a very similar response to the language stimulus. Acting in a unimanual manner makes this response slower, especially for the left-hand.

Activation of the right dorsal premotor cortex has been demonstrated both during unimanual [19] and bimanual [20] grasping tasks. However, the right dorsal premotor cortex activation during symmetrical bimanual grasping tasks does not exceed the sum of its activation during right and left-hand unimanual tasks [21]. The intensity of activation and, by extension, the influence of the right dorsal premotor cortex on the left M1 during both unimanual and symmetrical bimanual grasping tasks seems, therefore, to be the same. Also, it has been demonstrated [11] that the left M1 activation intensity is the same in similar unimanual and symmetrical bimanual activities.

The left supplementary motor area is left-lateralized in right-handed subjects since it can influence the right hand and the left hand in unimanual tasks [22, 23], whereas its contralateral homologue is active only when the left hand is performing. Both regions, however, are active when the upper limbs act in an asymmetrical or symmetrical bimanual way, making the supplementary motor area directly related to bimanual movement [21, 23]. Thus, the modulation of the right hand grip force relies on almost the same circuitry during unimanual and symmetrical bimanual tasks, but the supplementary motor area and its role in the upper limbs’ bimanual movement may support our results regarding the RT values for the right hand during unimanual and symmetrical bimanual tasks.

The micro-window analysis showed that the bimanual task RT for the left hand took place at the 180–190 ms micro-window. During symmetrical bimanual movement, the right dorsal premotor cortex has shown an activation level similar to the summation of its activation levels at the unimanual activity for each hand, which could indicate that this region does not have a specific role for the accomplishment of this movement [21]. The left hemisphere shared control of the bimanual task may account for the proximity of the RT between SBR and SBL. Broca’s area has access to the right M1, but since the right M1 shows locally driven activation, Broca’s area may only exert partial influence over it. On the other hand, Broca’s area has full influence over the left premotor cortex, thus holding a higher level of influence in a symmetrical bimanual condition.

Kelso et al. [24] found no significant difference between RTs for the right and the left hand during a bimanual symmetrical task. Although some studies have obtained values for the interhemispheric transfer time (IHTT) assessed by RT to be between 3 and 5 ms, and the IHTT assessed by the ERP from 10 to 20 ms [25–26], Jäncke et al. [27] stated that the IHTT lies between 15 and 35 ms. These authors, however, assessed the IHTT due to an auditory stimulus in a phoneme discrimination task, while other studies used visual stimuli. Thus, we can assume that the difference found between the RTs for the left and right hand during the bimanual symmetrical task in this study may be due to the IHTT. Our protocol does not allow us to arbitrate the influence of the IHTT or to distinguish that of the partially independent right M1 activation.

Regarding the left-hand in the unimanual task, the micro-window analysis showed that the unimanual task RT occurred within 320–330 ms. Given the simplicity of the task, three reasonable assumptions to explain this result are formulated:

### Broca’s area role

Broca’s area may account for the grip force modulation. To do so, Broca’s area must stimulate the left Brodmann area 6 to the point at which the latter is able to surmount the interhemispheric inhibition imposed by the right Brodmann area 6 for preventing undesirable symmetrical movements. That would explain the greater RT for the left hand as compared to the right hand. In a very recent study, five distinct sub-regions were mapped in the right premotor cortex [28]. Each sub-region has distinct connections, thus exerting different and distinct functions. Broca’s area would be able to influence the right premotor cortex by one or more of these sub-regions, thus being able to modulate left hand grip force without stimulating the right hand symmetrical movement.

### Broca’s homologue area role

Broca’s homologue area may solely account for the grip force modulation since it has the same connections in the right hemisphere that Broca’s area has in the left [8, 29]. The disruption of the right inferior frontal gyrus by means of the rTMS was found to induce naming errors in an fMRI study [30], demonstrating that this region exerts an active contribution to linguistic function. Jung-Beeman [31] divided the semantic processing into three components: integration, activation, and selection, the first occurring in the temporal cortex, the second in the parietal cortex, yet still relying on some temporal cortex portions, and the third in the inferior frontal gyrus. The author stated that they are inextricably linked, occurring bilaterally with little interhemispheric integration. According to this model, the Broca’s homologue area would account for the selection in the right hemisphere. The complexity of the linguistic processing required defines the activation intensity of Broca’s homologue area, and this intensity may influence the subject’s final response. Therefore, the greater RT for the left hand during a unimanual task could be due to the right hemisphere demanding a stronger level of activation, as discussed above.

### Bilateral control

Instead, a direct connection between Broca’s area and Broca’s homologue area may allow for both areas to be responsible for the grip force modulation. In this case, Broca’s area could stimulate its homologue such that it could ultimately stimulate the right premotor cortex for producing the grip force modulation observed. In a connectivity modeling meta-analysis study, Broca’s homologue area was found to be a relevant relay for Broca’s area [32]. Rosso et al. [29] found that inhibition of Broca’s homologue area by cathodal transcranial direct current stimulation (tDCS) was able to improve speed in a picture naming test. They found that this improvement was directly correlated to the frontal aslant tract (FAT) volume, which connects Broca’s homologue area to the right supplementary motor area. Therefore, a stronger connection between Broca’s homologue area and the right supplementary area could account for slower picture naming. These results could account for the greater RT for the left hand as they indicate that Broca’s homologue area’s information processing demands more time than its counterpart. The intrinsic characteristics of the regions of the right hemisphere engaged in language function, as well as the number of connections and the dendritic arborization, account for an overall slower linguistic processing [31]. Thus, the grip force modulation that we found in our study could be starting later because the internal processing taking place in Broca’s homologue area is naturally slower or because it needs a stronger stimulation to be activated.

Broca’s area’s engagement relies entirely on the classic view of left-hemisphere dominance, but collides with the problem of selective disruption of the interhemispheric inhibition that prevents undesirable symmetrical movements. Control of the left hand during a unimanual activity is exerted by the right supplementary motor area and the right dorsal premotor cortex as well as by the left supplementary motor area [22], and these connections may allow Broca’s area to access the right M1. However, the lack of influence by the left dorsal premotor cortex over the right M1 may render Broca’s area/the left supplementary motor area/the right M1 pathway unable to efficiently modulate the left-hand grip force. Since the right dorsal premotor cortex accounts for the right M1 activation of left hand movement, the left dorsal premotor cortex is unable to influence it during left unimanual tasks. Therefore, the drawback of the Broca’s area role is that Broca’s area should stimulate Brodmann area 6 without spoiling the undesirable symmetrical movement prevention.

Presuming that the interhemispheric inhibition completely blocks Broca’s area’s influence over the right M1, Broca’s homologue area could account for the observed grip force modulation, since it is somewhat active due to bilateral semantic processing. However, few articles could support this assumption until now. The bilateral control allows us to not rule out Broca’s area role control and is supported by more studies [31, 33].

Beyond these considerations, one must consider that the described circuits refer largely to the motor and linguistic processing engaged in voluntary action. Our study looked at the influence of the manual action verb on a voluntary motor action being executed. While this motor action can be described as voluntary and isometric, the linguistic influence occurs completely unintentionally. Since modulation of the force described here is not intended to produce a movement or correct the ongoing movement, the feedback mechanisms usually described as part of language processing and verbal production are not triggered here. Nevertheless, the observation that linguistic function modulates the bilateral grip force shows that the coupling of the motor system to the language system occurs extensively and is not limited to the left hemisphere. Although there is much discussion about the real influence of the supplementary motor area and premotor cortex on the ipsi- and contralateral M1, our study showed that listening to a word denoting a manual action produced a reaction that extended to both hemispheres and could be detected by the grip force exerted by each hand, either independently or jointly, in a symmetrical movement. We could consider that the premotor cortex and the supplementary area have been investigated as part of the mirror-neuron system in motor/language processing [34–36], and that their activation has been linked to visual and auditory stimuli, but our study does not allow us to make any inference about the influence of this system in our findings. More studies are required to determine the influence of these results on motor skill development or strength performance programs with normal or brain-damaged subjects.

## Supporting information

S1 Table Words used in this study. The action verbs and the non-action nouns in Portuguese and their equivalents in French and English.
(PDF)

S1 Study dataset A series of sheets containing the study data, organized by task and by hand. The data was grouped by the divisions described in the text (phases, time interval and micro-windows).
(XLSX)
