# Supplementary material for: Manual action verbs modulate the grip force of each hand in unimanual or symmetrical bimanual tasks
Source: PLoS One. 2018 Feb 5;13(2):e0192320. doi: 10.1371/journal.pone.0192320 (PMC5798821; doi:10.1371/journal.pone.0192320)
Supplement: S1 Table — The action verbs and the non-action nouns in Portuguese and their equivalents in French and English. (PDF) [file pone.0192320.s001.pdf]

Word list - translations

	Portuguese verbs	French verbs	English verbs
1	Abrir	Ouvrir	To open
2	Acariciar	Caresser	To fondle
3	Agarrar	Saisir	To grasp
4	Alisar	Lisser	To smooth
5	Amarrar	Attacher	To tie
6	Anotar	Annoter	To annotate
7	Apertar	Appuyer	To compress
8	Apontar	Pointer	To point
9	Bater	Battre	To beat
10	Beliscar	Pincer	To pinch
11	Botar	Mettre	To ser
12	Catar	Épouiller	To pick
13	Colocar	Placer	To place
14	Desenhar	Dessiner	To draw
15	Dirigir	Conduire	To drive
16	Escrever	Écrire	To write
17	Esfregar	Frotter	To rub
18	Estalar	Craquer	To snap
19	Fechar	Fermer	To close
20	Juntar	Joindre	To join
21	Lançar	Lancer	To throw
22	Lavar	Laver	To wash
23	Mexer	Mélanger	To stir
24	Misturar	Mélanger	To mix
25	Pegar	Prendre	To take
26	Pintar	Peintre	To paint
27	Por	Mettre	To place
28	Puxar	Tirer	To pull
29	Rabiscar	Griffonner	To scrabble
30	Receber	Recevoir	To receive
31	Retirar	Rétirer	To withdraw
32	Riscar	Risquer	To scratch
33	Segurar	Tenir	To hold
34	Soltar	Détacher	To drop
35	Tomar	Prendre	To seize
	**Portuguese nouns**	**French nouns**	**English nouns**
1	Antena	Antenne	Antenna
2	Aranha	Araignée	Spider
3	Avião	Avion	Avion

4	Mala	Bagage	Suitcase
5	Bola	Ballon	Ball
6	Bolsa	Bourse	Bag
7	Caderno	Cahier	Notebook
8	Pato	Canard	Duck
9	Sapato	Chaussure	Shoe
10	Repolho	Chou	Cabbage
11	Prego	Clou	Nail
12	Lanche	Colation	Snack
13	Domingo	Dimanche	Sunday
14	Dominó	Domino	Domino
15	Água	Eau	Water
16	Escola	École	School
17	Abraço	Embrassade	Hug
18	Escada	Escalier	Ladder
19	Estrela	Étoile	Star
20	Giló	Gilo	Giloh
21	Sapo	Grenouille	Frog
22	Brinquedo	Jouet	Toy
23	Leite	Lait	Milk
24	Mamãe	Mère	Mother
25	Relógio	Montre	Clock
26	Urso	Ours	Bear
27	Papai	Père	Father
28	Pé	Pied	Foot
29	Tábua	Planche	Board
30	Reunião	Réunion	Meeting
31	Mochila	Sac-à-dos	Schoolbag
32	Apito	Sifflet	Whistle
33	Rato	Souris	Mouse
34	Tatu	Tatou	Armadillo
35	Carro	Voiture	Car
